# The rs1458038 variant near *FGF5* is associated with poor response to calcium channel blockers among Filipinos

**DOI:** 10.1097/MD.0000000000028703

**Published:** 2022-02-04

**Authors:** Felix Eduardo R. Punzalan, Eva Maria C. Cutiongco – de la Paz, Jose Jr. B. Nevado, Jose Donato A. Magno, Deborah Ignacia D. Ona, Aimee Yvonne Criselle L. Aman, Marc Denver A. Tiongson, Elmer Jasper B. Llanes, Paul Ferdinand M. Reganit, Richard Henry P. Tiongco, Lourdes Ella G. Santos, Jaime Alfonso M. Aherrera, Lauro L. Abrahan, Charlene F. Agustin, Adrian John P. Bejarin, Rody G. Sy

**Affiliations:** a Department of Internal Medicine, University of the Philippines – Philippine General Hospital, Manila; b Institute of Human Genetics, National Institutes of Health, University of the Philippines, Manila; c Philippine Genome Center, University of the Philippines, Diliman, Quezon City, Manila.

**Keywords:** calcium channel blockers, fibroblast growth factor 5 gene, hypertension, rs1458038

## Abstract

Genetic variation is known to affect response to calcium channel blockers (CCBs) among different populations. This study aimed to determine the genetic variations associated with poor response to this class of antihypertensive drugs among Filipinos.

One hundred eighty one hypertensive participants on CCBs therapy were included in an unmatched case-control study. Genomic deoxyribonucleic acid were extracted and genotyped for selected genetic variants. Regression analysis was used to determine the association of genetic and clinical variables with poor response to medication.

The variant rs1458038 near *fibroblast growth factor 5 gene* showed significant association with poor blood pressure-lowering response based on additive effect (CT genotype: adjusted OR 3.41, *P* = .001; TT genotype: adjusted OR 6.72, *P* < .001).

These findings suggest that blood pressure response to calcium channels blockers among Filipinos with hypertension is associated with gene variant rs1458038 near *fibroblast growth factor 5 gene*. Further studies are recommended to validate such relationship of the variant to the CCB response.

## 1 Introduction

Hypertension continues to be a major health concern among Filipinos and worldwide. Left untreated, elevated blood pressure can lead to several fatal consequences such as chronic kidney disease, heart failure, myocardial infarction, and stroke. Despite availability of anti-hypertensive drugs, the global burden of hypertension remains substantial at 20,526 per 100,000 individuals with systolic BP of 140 mm Hg or higher.^[1]^ In the Philippines, as of 2018, an estimated 19.2% of the adult population have a blood pressure of 140/90 mm Hg or higher.^[2]^

Calcium channel blockers (CCBs) are among the firstline medications used to treat hypertension.^[3]^ These drugs prevent the contraction of vascular smooth muscle cells through blockade of calcium (Ca^2+^) ion influx, inducing vasodilation, and a decrease in total peripheral resistance, which lower blood pressure.^[4]^ Clinical trials have shown that CCBs are well-tolerated and effectively improve blood pressure, and the use of CCBs result to reduced major coronary events that is comparable with other drug classes.^[5–7]^ Their well-established efficacy as well as accessibility explain why CCBs have ranked as the second most prescribed drug for hypertension in the Philippines.^[8]^

The effectiveness of CCBs in lowering blood pressure has been investigated in terms of both ethnic differences and genetic polymorphisms. African Americans demonstrated better responses to CCBs compared to Caucasians.^[9]^ Among Asians, CCBs were reported to be more effective in controlling blood pressure and reducing risk for stroke compared with other drug classes.^[10]^ Pharmacogenetic studies showed that polymorphisms in calcium voltage-gated channel subunit alpha1 C (*CACNA1C*) are associated with CCB response among Caucasians,^[11]^ cytochrome P450 3A4 (*CYP3A4*) among African American,^[12]^ and urea transporter 2 *solute carrier family 14 member 2* among Chinese.^[13]^ However, outcomes from these studies cannot be generalized across populations due to inherent interpopulation differences in genetic profile. Thus, population-specific studies of candidate genes are needed to translate pharmacogenetic findings into clinical applications.

Studies done on candidate variants associated with hypertension and CCB response were done mostly on cohorts in which Filipinos were underrepresented. Hence, we determined the association of variants of selected genes with poor response to CCBs among Filipinos. Findings from this study can potentially identify markers for CCB use in the treatment of hypertension.

## 2 Methods

### 2.1 Enrollment of participants

Ambulatory, nonadmitted participants from the Philippine General Hospital outpatient clinics and volunteer staff, Metro Manila communities, and private clinics were enrolled from June 2013 to March 2017 in an unmatched case-control study investigating the association of candidate variants with poor response to CCBs. The following inclusion criteria were used:

1. 18 years or older;
2. able to independently provide consent;
3. of Filipino descent up to the 3rd degree of consanguinity;
4. diagnosed with hypertension according to the 7th Report of the Joint National Commission on Prevention, Detection, Evaluation and Treatment of High Blood Pressure (JNC 7); and
5. on CCB treatment (e.g., amlodipine, felodipine) for at least 1 month.

Participants were excluded if they had decompensated diseases of the lung or liver, heart failure, end-stage renal disease, active malignancy, secondary hypertension, secondary dyslipidemia, or were pregnant during the study period. Participants related to other enrolled participants up to 3rd degree of consanguinity were also excluded.

Participants are labeled as *CCB poor responders* (cases) if they still have high blood pressure (systolic BP greater than or equal to 140 mm Hg or diastolic BP greater than or equal to 90 mm Hg) on monitoring or on follow-up despite being on maximum dose of CCBs, either as monotherapy or as part of a multiple antihypertensive regimen. Participants are considered *CCB responders* (controls) if their blood pressure readings are less than 140/90 mm Hg on monitoring or on follow-up, while on CCB monotherapy. The blood pressure thresholds were based on the definition of hypertension in JNC7.

### 2.2 Sample size calculation

Sample sizes were computed in the presumed setting of a recessive model to provide the largest sample size estimate, a minimum minor allele frequency of 20% (MAF ≥ 0.20), and an alpha of 0.05, with a case-control ratio of 1:2. The minimum sample size per subgroup was set at 62 cases and 124 controls.

### 2.3 Clinical data collection

Researchers obtained demographic data and clinical characteristics of the participants such as age, sex, co-morbidities, smoking, and alcohol use status, and results of previous diagnostic tests from patient records and verbal interviews. Clinical chemistry tests were requested to obtain recent lipid profile and serum creatinine levels.

### 2.4 Deoxyribonucleic acid extraction and quantification

Deoxyribonucleic acid (DNA) extraction from blood buffy coat was done using the QiaAmp DNA minikit (QIAGEN, Victoria, Australia), following a spin protocol indicated in manufacturer's instruction manual. DNA was quantified using a spectrometer at 260 nm and stored at −20°C until use. All DNA samples had A-260 nm/A-280 nm value equal or above 1.80. These methods are similar to the methods done in studies published earlier by the authors.^[14,15]^

### 2.5 Genotyping

Ninety six candidate variants from both coding and noncoding regions which were associated with hypertension and calcium-channel blocker response were included in a customized GoldenGate Genotyping (GGGT) beadchip (Illumina, Inc., San Diego, CA) designed in 2012 (see Table S1, Supplemental Digital Content which describes the variants, and includes the studies/ patents from which they were referenced). Extensive searches were done in the following databases, looking at the risk and protective odds ratios (OR) of the variants: PharmGKB (Pharmacogenomics Knowledgebase) database, National Human Genome Research Institute Genome-Wide Association Study (GWAS) Catalog, PubMed, and selected patent databases (e.g., Patentscope and Espacenet). These variants were submitted to Illumina, Inc. for scoring to determine their suitability to be incorporated into the beadchip and to estimate their specificity.

Customized genotyping of candidate SNPs was performed using DNA microarray technology following the GoldenGate Genotyping protocol specified in the manufacturer's manual. After microarray processing, the beadchips were imaged on the HiScan System and data from these images were analyzed using GenomeStudio software. Variant selection and genotyping methods are similar to steps published in previous studies.^[14,15]^

### 2.6 Data analyses

*Quality control.* Genotype data from participants with call rates >95% upon evaluation with GenomeStudio version 2.0 and with missing genotype data in less than 5% of the SNPs (individual missingness test, *mind*) on PLINK version 2.05.10 were included. The following thresholds were also used in PLINK for inclusion of the genotype data: minor allele frequency of 0.01 (frequency test, MAF), with missing genotype in less than 5% of the individuals (genotype missingness test, *geno*), and a *P* value < .001 on Hardy–Weinberg Equilibrium (HWE) test among controls.

### 2.7 Statistical analysis

Chi-Squared test (for categorical data) and Student *t* test (for quantitative data) were used to compare the 2 groups based on clinical and demographic qualities. No imputations were made for missing data; best-case/worst-case scenario analysis was done to determine whether there is significant difference between the case and control groups based on the proportion of participants with elevated BMI. Fisher exact tests were performed to assess for significant differences between alleles (allelic association tests) and genotypes (genotypic association tests). The most likely genotypic model – dominant, recessive, or additive – was also determined based on the distribution of the genotypes among cases and controls. Variants were selected if the *P* value of these tests are less than the Bonferroni-corrected α adjusted for multiple testing. Genotypes are recoded based on the significant genotypic model in preparation for univariate analysis using Stata 14.0. Univariate logistic regression analysis was done to determine the OR of the variant-CCB response association (α = 0.05). Multiple logistic regression was performed to assess the effect of possible confounding clinical factors on the association of the variants, and variable selection using backward elimination was done to determine which among the variables are most likely to be associated with poor response to CCBs.

Quality control and statistical analyses were similar to the methods done in studies published earlier by the authors.^[14,15]^

### 2.8 Ethical considerations

The University of the Philippines Manila - Research Ethics Board approved all procedures of the study in compliance with its ethical standards (Study protocol code UPMREB-2012-0186-NIH, approval date April 10, 2012).

## 3 Results

**Figure 1:**
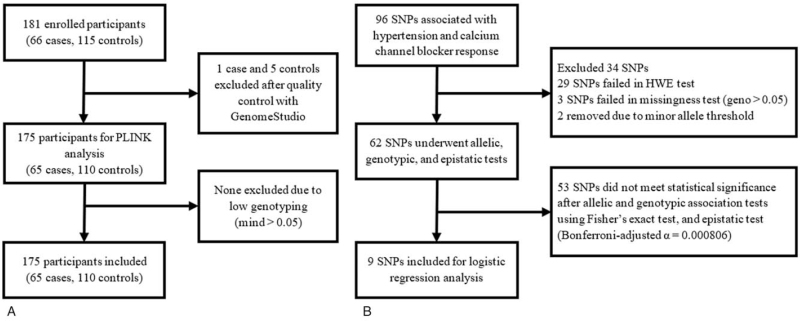
Overview of data processing and analysis. geno = genotypic missingness, HWE = Hardy-Weinberg equilibrium, MAF = minor allele frequency, mind = individual missingness, SNP = single nucleotide polymorphism.

A total of 181 participants were enrolled in the study. One hundred seventy five participants (65 cases and 110 controls) remained after quality control (Fig. 1A). Sixty two variants remained from the 96 SNPs (Fig. 1B). Data was still analyzed as planned, despite a lower turnout of controls than computed.

Comparison of baseline characteristics between cases and controls is summarized in Table 1. There were no significant differences in age and sex between the 2 groups. There were more patients with diabetes among the poor responders to CCB. Twelve participants (6 responders, 6 nonresponders) did not have body mass index data; however, there was still no significant difference between the 2 groups after further analysis (see Table S2, Supplemental Digital Content which shows the results of the best-case/ worst-case analysis based on elevated BMI). There was significantly higher percentage of participants with ischemic heart disease among poor responders to CCB than responders (24.62% vs 5.50%, *P* < .001). Poor responders to CCB also seemed to have slightly elevated creatinine but with similar lipid profile compared to responders.

**Table 1:** Clinical characteristics of study participants.

Characteristics	Poor responders to CCB (n = 65)	Responders to CCB (n = 110)	*P* value^∗^
Age in years, mean (SD)	59 (12)	58 (9)	NS
Male sex, %	43.08	40.00	NS
Calcium channel blocker used, %			NS
Amlodipine	98.46	99.09	
Nifedipine	1.54	0.91	
Dyslipidemia, %	80.00	69.09	NS
Type 2 diabetes mellitus, %	43.08	21.82	.003
Abnormal BMI (≥ 25 kg/m^2^), %	52.54	47.12	NS
Ischemic heart disease, %	24.62	5.50	<.001
Stroke, %	24.62	15.45	NS
Lifestyle factors, %			
Smoking	40.00	30.00	NS
Alcohol drinking	60.00	50.91	NS
Creatinine in mg/dl, mean (SD)	0.98 (0.40)	0.83 (0.33)	.008
eGFR in ml/min, mean (SD)	81.92 (27.88)	93.27 (23.47)	.005
Total cholesterol in mg/dl, mean (SD)	204.21 (50.68)	201.46 (49.82)	NS
Triglycerides in mg/dl, mean (SD)	139.80 (76.64)	122.43 (64.20)	NS
HDL in mg/dl, mean (SD)	52.49 (19.70)	51.58 (12.53)	NS
LDL in mg/dl, mean (SD)	124.25 (44.91)	127.45 (40.58)	NS

N = 175. BMI = body mass index, CCB = calcium channel blocker, eGFR = estimated glomerular filtration rate, HDL = high-density lipoprotein, IHD = ischemic heart disease, LDL = low-density lipoprotein, NS = not significant, SD = standard deviation.

∗ Significance set at *P* < .05 using either Chi-Squared test or *t* test.

### 3.1 Logistic regression analyses

There were 10 variants found to have statistically significant association with poor response to CCBs on genotypic association test after adjusting for multiple testing (Bonferroni-adjusted α = 8.06X10^-4^) (see Table S3, Supplemental Digital Content list of variants included after genotypic association tests). One SNP, rs991316, was excluded after epistasis test; the rest of the variants underwent logistic regression analysis to compute for their OR and to determine which will retain significance upon adjusting for other variables (Table 2). Two SNPs had clinically significant odds ratios (OR ≥ 2.5) and were statistically significant (*P* < .001) on simple logistic regression analysis: rs1458038 in *FGF5*, and rs776746 in *CYP3A5*. Both SNPs seemed to display an additive genotypic effect.

**Table 2:** Univariate logistic regression of 9 genetic variants.

					Allele frequencies, %^∗^		Genotypic frequencies, %^∗^						
Variants	Chr	Gene	Variant role	Risk allele (A)	A	a	AA	Aa	aa	Genotype	Crude OR (95% CI)		*P* value^∗∗^
rs12046278	1	*CASZ1*	intron variant	T	40.77	59.23	18.46	44.62	36.92	TC vs CC	2.45	(1.24, 4.84)	.010
					21.82	78.18	6.36	30.91	62.73	TT vs CC	4.93	(1.74, 13.96)	.003
rs13420028	1	*GPR39*	intron variant	T	87.69	12.31	76.92	21.54	1.54	TG vs GG	6.80	(0.82, 56.08)	.075
					68.64	31.36	52.73	31.82	15.45	TT vs GG	14.66	(1.88, 114.06)	.010
rs1458038	4	*FGF5*	intergenic	T	49.23	50.77	27.69	43.08	29.23	TC vs CC	3.66	(1.77, 7.55)	<.001
					21.36	78.64	8.18	26.36	65.45	TT vs CC	7.58	(2.94, 19.53)	<.001
rs16982520	20	*ZNF831*	intron variant	A	91.54	8.46	86.15	10.77	3.08	AG vs GG	1.45	(0.26, 8.00)	.671
					75.91	24.09	62.73	26.36	10.91	AA vs GG	4.87	(1.05, 22.67)	.044
rs653178	12	*ATXN2*	intron variant	A	86.92	13.08	81.54	10.77	7.69	AA vs AG/GG	3.55	(1.71, 7.37)	.001
					70.91	29.09	55.45	30.91	13.64				
rs776746	7	*CYP3A5*	splice acceptor	G	66.92	33.08	47.69	38.46	13.85	AG vs AA	3.44	(1.43, 8.23)	.006
					40.00	60.00	22.73	34.55	42.73	GG vs AA	6.48	(2.67, 15.72)	< .001
rs9350602	6	*MYO6*	intron variant	C	90.77	9.23	84.62	12.31	3.08	TC vs TT	1.87	(0.35, 9.96)	.465
					73.64	26.36	60.00	27.27	12.73	CC vs TT	5.83	(1.27, 26.78)	.023
rs11780975	8	*HSPE1P14*	intergenic	C	96.92	3.08	93.85	6.15	0.00	AC vs AA	0.48	(0.04, +inf)	> .999
					84.09	15.91	70.91	26.36	2.73	CC vs AA	2.95	(0.31, +inf)	.365
rs1799945	6	*HFE*	missense	C	96.15	3.85	92.31	7.69	0.00	GC vs GG	2.24	(0.27, +inf)	.497
					81.82	17.27	71.82	20.00	7.27	CC vs GG	8.22	(1.23, +inf)	.026

a = non-risk allele, A = risk allele, ATXN2 = ataxin 2, CASZ1 = castor zinc finger 1, Chr = chromosome, CI = confidence interval, CYP3A5 = cytochrome P450 family 3 subfamily A member 5, FGF5 = fibroblast growth factor 5, GPR39 = G protein-coupled receptor 39, MYO6 = myosin VI, OR = odds ratio, ZNF831 = zinc finger protein 831. All variants exhibit an additive model of inheritance, except for rs653178.

∗ The upper values in the frequency columns are frequencies among cases, while the lower values are frequencies among controls.

∗∗ Significance set at *P* < .05 on simple univariate logistic regression analysis, except for rs11780975 and rs1799945 which were analyzed using exact logistic regression analysis due to empty cells.

Simple logistic regression was also done for the clinical variables (Table 3). Factors including type 2 diabetes mellitus, dyslipidemia, and smoking (*P* < .2) were included together with the 7 variants in the multiple regression analysis (see Table 4), genetic variants and clinical factors in the multiple regression full model). On variable selection through backward elimination (*P* < .05), only the presence of DM and rs1458038 retained their association in relation to poor response to CCB (Table 5). Participants who had the risk allele T was associated with poor response to CCBs, with 3.41 times higher odds among heterozygotes (TC) and 6.72 times higher odds among homozygotes (TT). This doubling in odds clearly demonstrated the additive effect described for this variant.

**Table 3:** Simple logistic regression of significant clinical factors.

Clinical Factors	Frequency in cases, % (n)	Frequency in controls, % (n)	Crude OR	(95% CI)	*P* value
Age ≥ 60 years	49.23 (32)	43.64 (48)	1.25	(0.68, 2.32)	.473
Male sex	43.08 (28)	40.00 (44)	1.13	(0.61,2.11)	.689
Dyslipidemia	80.00 (52)	69.09 (76)	1.79	(0.86, 3.71)	.118
Type 2 diabetes mellitus	43.08 (28)	21.82 (24)	2.71	(1.39, 5.29)	.003
Ischemic heart disease	24.62 (16)	5.50 (6)	5.61	(2.06, 15.21)	.001
Stroke	24.62 (16)	15.45 (17)	1.78	(0.83, 3.84)	.137
Lifestyle Factors					
Smoking	40.00 (26)	30.00 (33)	1.56	(0.82, 2.96)	.178
Alcohol	60.00 (39)	50.91 (56)	1.45	(0.78, 2.70)	.244

BMI = body mass index, CI = confidence interval, IHD = ischemic heart disease, OR = odds ratio.

**Table 4:** Genetic variants and clinical factors in the multiple regression full model.

Factors	Frequency among cases, % (n) N = 65	Frequency among controls, % (n) N = 110	Adjusted odds ratio	95% CI	*P* > z
Dyslipidemia	80.00 (52)	69.09 (76)	1.45	(0.57, 3.69)	.439
Type 2 DM	43.08 (28)	21.82 (24)	1.99	(0.84, 4.67)	.116
Smoking	40.00 (26)	30.00 (33)	1.24	(0.58, 2.69)	.579
rs12046278 (T)				(0.72, 8.39)	.151
TT	18.46 (12)	6.36 (7)	2.46 (TT vs CC)		
TC	44.62 (29)	30.91 (34)	1.43 (TC vs CC)	(0.63, 3.25)	.396
CC	36.92 (24)	62.73 (69)			
rs13420028 (T)				(0.73, 62.96)	.92
TT	76.92 (50)	52.73 (58)	6.79 (TT vs GG)		
TG	21.54 (14)	31.82 (35)	5.45 (TG vs GG)	(0.58, 51.39)	.138
GG	1.54 (1)	15.45 (17)			
rs1458038 (T)				(0.77, 10.11)	.120
TT	27.69 (18)	8.18 (9)	2.78 (TT vs CC)		
TC	43.08 (28)	26.36 (29)	1.61 (TC vs CC)	(0.54, 4.84)	.396
CC	29.23 (19)	65.45 (72)			
rs16982520 (A)				(0.20, 7.19)	.850
AA	86.15 (56)	62.73 (69)	1.19 (AA vs GG)		
AG	10.77 (7)	26.36 (29)	1.39 (AG vs GG)	(0.22, 8.79)	.726
GG	3.08 (2)	10.91 (12)			
rs653178 (A)				(0.18, 2.77)	.608
AA	81.54 (53)	55.45 (61)	0.70 (AA vs AG and GG)		
AG and GG	18.46 (12)	44.55 (49)			
rs776746 (G)				(0.87, 9.13)	.084
GG	47.69 (31)	22.73 (25)	2.82 (GG vs AA)		
AG	38.46 (25)	34.55 (38)	1.68 (AG vs AA)	(0.56, 5.03)	.351
AA	13.85 (9)	42.73 (47)			
rs9350602 (C)				(0.33, 12.13)	.445
CC	84.62 (55)	60.00 (66)	2.01 (CC vs TT)		
TC	12.31 (8)	27.27 (30)	1.42 (TC vs TT)	(0.23, 8.82)	.707
TT	3.08 (2)	12.73 (14)			

BMI = body mass index, CI = confidence interval.

**Table 5:** Genetic variants and clinical factors remaining after variable selection.

Factors	Frequency in cases, % (n)	Frequency in controls, % (n)	Adjusted OR (95% CI)	*P* value^∗^
rs1458038 (T)				< .001
TT	27.69 (18)	8.18 (9)	6.72 (2.56, 17.58) (TT vs CC)	
TC	43.08 (28)	26.36 (29)	3.41 (1.63, 7.11) (TC vs CC)	.001
CC	29.23 (19)	65.45 (72)		
Type 2 DM			2.23 (1.09, 4.55)	.028

CI = confidence interval, OR = odds ratio.

∗ Significance set at *P* < .05.

## 4 Discussion

Calcium channel blockers (CCBs) are widely-utilized in the armamentarium of drugs used to control hypertension. We determined candidate variants associated with CCB poor response on the background of clinical correlates. Together with type 2 diabetes mellitus (T2DM), single nucleotide polymorphism (SNP) rs1458038 shows the most robust association with poor response to CCBs among Filipinos.

The SNP rs1458038 is a 5’ upstream variant located in chromosome 4q21, 23 kb upstream of *fibroblast growth factor 5* (*FGF5)*. *FGF5* codes for a member of the fibroblast growth factor family of mitogenic proteins involved in cell differentiation, tissue repair, angiogenesis, and tumor growth.^[16]^ It is not yet known whether the variant exhibits a regulatory function on *FGF5* expression. The risk allele (T) frequency of the variant among the study participants is 21%. This T allele frequency is lower compared with East Asians (39%), admixed Americans (27%), Europeans (27%), and South Asians (25%), and much higher than Africans (4%).^[17]^

Although there have been no prior associations with poor CCB response, the variant rs1458038 has been associated with hypertension in general. This was shown in GWA studies involving European,^[18]^ Chinese,^[19]^ East African,^[20]^ and Japanese^[21]^ populations suggesting universal effect of the variant across ethnicities. Evidence for a hypertensive effect of FGFs is shown in murine studies wherein transgenic mice with enhanced FGF signaling exhibited higher mean arterial pressures.^[22]^ In humans, FGF signaling has been implicated in the development of pulmonary arterial hypertension.^[23]^

It is speculated that the poor CCB response among participants carrying the risk allele (T) of rs1458038 results to an upregulation of *FGF5* expression which impacts vascular tone. The effect of FGFs on vascular tone and high blood pressure possibly relies on a convergence with angiotensin II pathways. This has also been demonstrated in the previous study wherein the rise in blood pressure in the FGF signaling-enhanced mice was reversed by an angiotensin-receptor blocker (candesartan). Furthermore, it was shown through direct observations of cremaster and renal afferent arteriole diameters in mice that angiotensin II vasoconstriction was reversed by an FGF receptor kinase inhibitor.^[22]^

The modulation of intracellular calcium concentrations may be a linking mechanism for signaling of FGF, angiotensin II, and the influence of CCB on blood pressure. CCBs have been shown to impact Ca^2+^-dependent signaling mechanisms in vascular smooth muscle cells. This is evidenced in murine studies wherein the administration of amlodipine inhibited FGF-induced proliferation of vascular smooth muscle cells.^[24]^ Consequently, the crosstalk of FGF and angiotensin II is mediated by pathways that rely on an increase in cytosolic free Ca^2+^ concentrations such as mitogen-activated protein kinase (MAPK).^[22]^ Further studies can be done to investigate how these different pathways influence CCB control of blood pressure.

T2DM seems to be a confounder in this study, as it seems to be associated with both the SNP and the poor response outcome. Consistent with other studies, hypertensive patients with diabetes are the most resistant to treatment requiring two or more antihypertensive medications.^[25,26]^ It is postulated that several mechanisms contribute to the poor response to treatment for hypertension of patients with diabetes. These mechanisms include inappropriate activation of the renin angiotensin aldosterone system (RAAS), oxidative stress brought production of reactive oxygen species leading to endothelial dysfunction and impaired vasodilation.^[27]^ Therefore, there is often an activated renin-angiotensin-aldosterone system, in coexistent diabetes and hypertension that can impair responsiveness of blood pressure control to CCB.

The study is subject to the inherent limitations of a case-control design. As such, prevalence data, likelihood ratios, and effect sizes of associated factors cannot be deduced. These statistics are important in determining the predictive value of the variant with regards to poor CCB response. It is recommended that future investigations use appropriate study designs towards the development of accurate genotypic measures. In addition, the mechanism of the variant behind the risk of CCB poor response remains to be further elucidated. Lastly, due to the possible convergence of FGF and angiotensin II pathways, it is recommended that the association of the variant to angiotensin-receptor blockers also be explored.

The study was also limited by the low sample size, given that there were several variants tested. The study aimed to confirm whether variants that were previously associated with hypertension and response to calcium channel blockers (CCBs) among other populations are likely to be associated with poor response to CCBs among Filipinos as well, so these were the variants selected and included in the array. The sample size requirement was computed per individual SNP, with the goal of identifying whether these variants would retain at least nominal statistical significance. Statistical power calculation was done as suggested, using Genetic Power Calculator (see Table S4, Supplemental Digital Content, which shows the required number of cases and controls for an alpha of 0.00081 and power of 80%). Looking at the alpha which was set a priori, the study was able to reach a statistical power of 84%. This calculation is deemed sufficient for the purpose of initial exploration and screening. Nonetheless, if we are to consider the adjusted alpha after Bonferroni correction, the power is reduced to 35%. It is then highly recommended by the group that the study be validated in an independent population with at least 124 cases and 282 controls.

In conclusion, responses to certain drugs are found to be influenced by genetic variation among different populations. This study has shown that poor blood pressure response to CCBs among Filipinos with hypertension appears to be associated with the variant rs1458038 near *FGF5*. Further studies are being planned to validate current findings for possible application to individualized treatment of hypertension with CCBs.

## Acknowledgments

We thank Dr. Ivy Melgarejo, Dr. Winston Li, and Dr. Julius Gatmaitan, Virginia dela Cruz, Ralph Duhaylungsod, Romer Guerbo, Michael Hernandez, Hazel Joyohoy, Jessa Lu, Babylyn Pernites, Angela Ramones, Keith Serrano, Jonathan Terante, Ralph Torres, and Pauline Villanueva for recruiting patients. We also thank Chembie Almazar, Jessica Biwang, Roemel Jeusep Bueno, Reynand Canoy, Mae Belle Lacson, Jodelyn Melegrito, and Kate Wad-Asen of the Microarray Unit of the Institute of Human Genetics, National Institutes of Health for assistance in genotyping and research administration, and Prof. Cynthia Cordero and Prof. Kim Cochon for consultation with statistical analysis. Lastly, we thank Ms. Cheenee Calantoc, Dr. Jan Cyril Hiwatig, Dr. Alvin Lirio, Dr. Maria Bettina Quiambao, Dr. Gladys Catibog, and Dr. Samantha Llamzon for assisting with the writing of this manuscript.

## Author contributions

**Conceptualization:** Felix Eduardo Rubia Punzalan, Eva Maria Cruz Cutiongco - de la Paz, Jose Jr. Bautista Nevado, Jose Donato Acuña Magno, Deborah Ignacia David Ona, Elmer Jasper Balasico Llanes, Paul Ferdinand Mancera Reganit, Richard Henry Perlas Tiongco, Lourdes Ella Gonzalez Santos, Rody Gan Sy.

**Data curation:** Felix Eduardo Rubia Punzalan, Eva Maria Cruz Cutiongco - de la Paz, Jose Jr. Bautista Nevado, Jose Donato Acuña Magno, Deborah Ignacia David Ona, Aimee Yvonne Criselle Landicho Aman, Marc Denver Aquino Tiongson, Elmer Jasper Balasico Llanes, Paul Ferdinand Mancera Reganit, Richard Henry Perlas Tiongco, Lourdes Ella Gonzalez Santos, Jaime Alfonso Manalo Aherrera, Lauro IV Lim Abrahan, Charlene Francisco Agustin, Adrian John Pabrua Bejarin, Rody Gan Sy.

**Formal analysis:** Jose Jr. Bautista Nevado, Aimee Yvonne Criselle Landicho Aman, Adrian John Pabrua Bejarin.

**Funding acquisition:** Eva Maria Cruz Cutiongco - de la Paz, Rody Gan Sy.

**Investigation:** Felix Eduardo Rubia Punzalan, Eva Maria Cruz Cutiongco - de la Paz, Jose Jr. Bautista Nevado, Jose Donato Acuña Magno, Deborah Ignacia David Ona, Aimee Yvonne Criselle Landicho Aman, Marc Denver Aquino Tiongson, Elmer Jasper Balasico Llanes, Paul Ferdinand Mancera Reganit, Richard Henry Perlas Tiongco, Lourdes Ella Gonzalez Santos, Jaime Alfonso Manalo Aherrera, Lauro IV Lim Abrahan, Charlene Francisco Agustin, Adrian John Pabrua Bejarin, Rody Gan Sy.

**Methodology:** Felix Eduardo Rubia Punzalan, Eva Maria Cruz Cutiongco - de la Paz, Jose Jr. Bautista Nevado, Jose Donato Acuña Magno, Deborah Ignacia David Ona, Elmer Jasper Balasico Llanes, Paul Ferdinand Mancera Reganit, Richard Henry Perlas Tiongco, Lourdes Ella Gonzalez Santos, Rody Gan Sy.

**Project administration:** Eva Maria Cruz Cutiongco - de la Paz, Jose Jr. Bautista Nevado, Aimee Yvonne Criselle Landicho Aman, Rody Gan Sy.

**Resources:** Eva Maria Cruz Cutiongco - de la Paz, Jose Jr. Bautista Nevado, Aimee Yvonne Criselle Landicho Aman, Rody Gan Sy.

**Supervision:** Felix Eduardo Rubia Punzalan, Eva Maria Cruz Cutiongco - de la Paz, Jose Jr. Bautista Nevado, Jose Donato Acuña Magno, Deborah Ignacia David ONA, Elmer Jasper Balasico Llanes, Paul Ferdinand Mancera REGANIT, Richard Henry Perlas Tiongco, Lourdes Ella Gonzalez Santos, Rody Gan Sy.

**Visualization:** Felix Eduardo Rubia Punzalan, Jose Jr. Bautista Nevado, Aimee Yvonne Criselle Landicho Aman, Marc Denver Aquino Tiongson, Adrian John Pabrua Bejarin.

**Writing – original draft:** Felix Eduardo Rubia Punzalan, Jose Jr. Bautista Nevado, Aimee Yvonne Criselle Landicho Aman, Marc Denver Aquino Tiongson, Adrian John Pabrua Bejarin.

**Writing – review & editing:** Felix Eduardo Rubia Punzalan, Eva Maria Cruz Cutiongco - de la Paz, Jose Jr. Bautista Nevado, Jose Donato Acuña Magno, Deborah Ignacia David Ona, Aimee Yvonne Criselle Landicho Aman, Marc Denver Aquino Tiongson, Elmer Jasper Balasico Llanes, Paul Ferdinand Mancera Reganit, Richard Henry Perlas Tiongco, Lourdes Ella Gonzalez Santos, Jaime Alfonso Manalo Aherrera, Lauro IV Lim Abrahan, Charlene Francisco Agustin, Adrian John Pabrua Bejarin, Rody Gan Sy.

## Supplementary Material

Supplemental Digital Content

## Supplementary Material

Supplemental Digital Content

## Supplementary Material

Supplemental Digital Content

## Supplementary Material

Supplemental Digital Content
