# Supplementary material for: The rs1458038 variant near FGF5 is associated with poor response to calcium channel blockers among Filipinos
Source: Medicine (Baltimore). 2022 Feb 4;101(5):e28703. doi: 10.1097/MD.0000000000028703 (PMC8812666; doi:10.1097/MD.0000000000028703)
Supplement: Supplemental Digital Content [file medi-101-e28703-s001.docx]

**Supplemental Table 1.** A table of 96 variants selected for their association with hypertension and CCB response

| ***SNP*** | ***Gene*** | ***Chr*** | ***Genetic effect*** | ***Clinical/ Phenotypic effect*** | ***Reference*** |
| --- | --- | --- | --- | --- | --- |
| rs10021303 | *BMPR1B* | 4 | intron variant | hypertension susceptibility | PLoS One. 2012;7(2):e31489 |
| rs1004467 | *CYP17A1* | 10 | intron variant | systolic hypertension susceptibility | Nat Genet. 2009 Jun;41(6):677-87 |
| rs10188442 | *GPR39* | 2 | intron variant | hypertension susceptibility | Hum Genet. 2011 Dec;130(6):725-33 |
| rs1024323 | *GRK4* | 4 | missense variant | hypertension susceptibility | Clin Chem. 2002 Dec;48(12):2131-40 |
| rs10492602 | intergenic | 13 | unknown | hypertension susceptibility | US20090155230 |
| rs10503669 | intergenic | 8 | unknown | hypertension susceptibility;  HDL level; triglyceride level | Nature. 2007 Jun 7;447(7145):661-78;  Nat Genet. 2011 Sep 11;43(10):990-5 |
| rs11014166 | *CACNB2* | 10 | intron variant | hypertension susceptibility | Nat Genet. 2009 Jun;41(6):677-87 |
| rs11024074 | *PLEKHA7* | 11 | intron variant | diastolic hypertension susceptibility | Nat Genet. 2009 Jun;41(6):677-87 |
| rs1110183 | intergenic | 9 | unknown | hypertension susceptibility | PLoS One. 2012;7(2):e31489 |
| rs11191548 | *CNNM2/**NT5C2* | 10 | downstream gene variant | systolic hypertension susceptibility | Nature. 2011 Sep 11;478(7367):103-9 |
| rs1137617 | *KCNH2* | 7 | stop gained | CCB response | PLoS One. 2013 Apr 22;8(4):e61317 |
| rs11646213 | intergenic | 16 | unknown | hypertension susceptibility | Hum Mol Genet. 2009 Jun 15;18(12):2288-96 |
| rs1173771 | intergenic | 5 | unknown | systolic hypertension susceptibility | Nature. 2011 Sep 11;478(7367):103-12 |
| rs11780975 | intergenic | 8 | unknown | hypertension susceptibility | US20090155230 |
| rs11823543 | *ZNF259/BUD13* | 11 | downstream gene variant | hypertension susceptibility; triglyceride level | Diabetes. 2011 Apr;60(4):1329-39 |
| rs11825181 | *BUD13* | 1 | intron variant | hypertension susceptibility; triglyceride level | Diabetes. 2011 Apr;60(4):1329-39 |
| rs12046278 | *CASZ1* | 1 | intron variant | systolic hypertension susceptibility | Nat Genet. 2009 Jun;41(6):677-87 |
| rs12522034 | intergenic | 5 | unknown | hypertension susceptibility | Hum Genet. 2011 Dec;130(6):725-33 |
| rs12653539 | intergenic | 5 | unknown | hypertension susceptibility | US20090155230 |
| rs12946454 | *PLCD3* | 17 | intron variant | systolic hypertension susceptibility | Nat Genet. 2009 Jun;41(6):666-76 |
| rs13052628 | intergenic | 21 | unknown | hypertension susceptibility | US20090155230 |
| rs13107325 | *SLC39A8* | 4 | missense variant | systolic hypertension susceptibility diastolic hypertension susceptibility | Nature. 2011 Sep 11;478(7367):103-12 |
| rs1327235 | intergenic | 20 | unknown | diastolic hypertension susceptibility | Nature. 2011 Sep 11;478(7367):103-9 |
| rs13333226 | *UMOD* | 16 | upstream gene variant | hypertension susceptibility | PLoS Genet. 2010 Oct 28;6(10):e1001177 |
| rs13420028 | *GPR39* | 2 | intron variant | hypertension susceptibility | Hum Genet. 2011 Dec;130(6):725-33 |
| rs1372662 | *ZFAT* | 8 | intron variant | hypertension susceptibility | Hum Genet. 2011 Dec;130(6):725-33 |
| rs1378942 | *CSK* | 15 | intron variant | systolic hypertension susceptibility diastolic hypertension susceptibility | Nature. 2011 Sep 11;478(7367):103-10;  Nat Genet. 2009 Jun;41(6):666-76 |
| rs1384394 | intergenic | 2 | unknown | hypertension susceptibility | US20090155230 |
| rs1458038 | intergenic | 4 | unknown | systolic hypertension susceptibility diastolic hypertension susceptibility | Nature. 2011 Sep 11;478(7367):103-9 |
| rs1461656 | *MYLK4* | 6 | intron variant | hypertension susceptibility | US20090155230 |
| rs15285 | *LPL* | 8 | 3' UTR variant | hypertension susceptibility; triglyceride level | Diabetes. 2011 Apr;60(4):1329-39 |
| rs1530440 | *C10orf107* | 10 | intron variant | diastolic hypertension susceptibility | Nat Genet. 2009 Jun;41(6):666-76 |
| rs1550576 | intergenic | 15 | unknown | hypertension susceptibility | PLoS Genet. 2009 Jul;5(7):e1000564 |
| rs16931920 | intergenic | 9 | unknown | hypertension susceptibility | US20090155230 |
| rs16948048 | *ZNF652* | 17 | upstream gene variant | diastolic hypertension susceptibility | Nat Genet. 2009 Jun;41(6):666-76 |
| rs16982520 | *ZNF831* | 20 | upstream gene variant | hypertension susceptibility | Nat Genet. 2009 Jun;41(6):677-87 |
| rs16998073 | *FGF5* | 4 | upstream gene variant | diastolic hypertension susceptibility | Nat Genet. 2009 Jun;41(6):666-76 |
| rs17367504 | *MTHFR* | 1 | missense variant | systolic hypertension susceptibility | Nat Genet. 2009 Jun;41(6):666-76 |
| rs17403547 | intergenic | 2 | unknown | hypertension susceptibility | US20090155230 |
| rs17589290 | intergenic | 4 | unknown | hypertension susceptibility | PLoS Genet. 2011 Feb 10;7(2):e1001300 |
| rs17608766 | *GOSR2* | 17 | 3' UTR variant | systolic hypertension susceptibility | Nature. 2011 Sep 11;478(7367):103-14 |
| rs1799945 | *HFE* | 6 | missense variant | systolic hypertension susceptibility diastolic hypertension susceptibility | Nature. 2011 Sep 11;478(7367):103-13 |
| rs1801058 | *GRK4* | 4 | missense variant | hypertension susceptibility | Clin Chem. 2002 Dec;48(12):2131-40 |
| rs1918974 | *MECOM* | 3 | intron variant | diastolic hypertension susceptibility | Nat Genet. 2009 Jun;41(6):666-77 |
| rs2070762 | *TH* | 11 | intron variant | hypertension susceptibility | CN1891822 |
| rs2246709 | *CYP3A4* | 7 | intron variant | CCB response | Am J Nephrol. 2010;31(2):95-103 |
| rs2384550 | intergenic | 12 | unknown | diastolic hypertension susceptibility | Nat Genet. 2009 Jun;41(6):677-87 |
| rs2398162 | *NR2F2-AS1* | 15 | intron variant | hypertension susceptibility | Nature. 2007 Jun 7;447(7145):661-78 |
| rs2469997 | intergenic | 8 | unknown | hypertension susceptibility | Hum Genet. 2011 Dec;130(6):725-33 |
| rs2521501 | *FES* | 15 | intron variant | systolic hypertension susceptibility diastolic hypertension susceptibility | Nature. 2011 Sep 11;478(7367):103-11 |
| rs2681472 | *ATP2B1* | 12 | intron variant | hypertension susceptibility diastolic hypertension susceptibility | Nat Genet. 2009 Jun;41(6):677-87 |
| rs2681492 | *ATP2B1* | 12 | intron variant | systolic hypertension susceptibility | Nat Genet. 2009 Jun;41(6):677-87 |
| rs2740574 | *CYP3A4* | 7 | upstream gene variant | CCB response | Am J Nephrol. 2010;31(2):95-103 |
| rs2820037 | intergenic | 1 | unknown | hypertension susceptibility | Nature. 2007 Jun 7;447(7145):661-78 |
| rs2932538 | *MOV10/**CAPZA1* | 1 | downstream gene variant | hypertension susceptibility | Nature. 2011 Sep 11;478(7367):103-9 |
| rs2954033 | intergenic | 8 | unknown | hypertension susceptibility; triglyceride level | Diabetes. 2011 Apr;60(4):1329-39 |
| rs2960306 | *GRK4* | 4 | missense variant | hypertension susceptibility | Clin Chem. 2002 Dec;48(12):2131-40 |
| rs3184504 | *SH2B3* | 12 | missense variant | systolic hypertension susceptibility diastolic hypertension susceptibility | Nat Genet. 2009 Jun;41(6):677-87 |
| rs32790 | intergenic | 5 | unknown | hypertension susceptibility | US20090155230 |
| rs36217263 | *KL* | 13 | upstream gene variant | hypertension susceptibility | KR1020130027093 |
| rs3798440 | *MYO6* | 6 | intron variant | hypertension susceptibility | Hum Genet. 2011 Dec;130(6):725-33 |
| rs381815 | *PLEKHA7* | 11 | intron variant | systolic hypertension susceptibility | Nat Genet. 2009 Jun;41(6):677-87 |
| rs4290 | *ACE* | 17 | upstream gene variant | hypertension susceptibility coronary outcome | Clin Pharmacol Ther. 2009 Jan;85(1):36-44 |
| rs448378 | *MECOM* | 3 | intron variant | systolic hypertension susceptibility | Nat Genet. 2009 Jun;41(6):677-87 |
| rs4686599 | intergenic | 3 | unknown | hypertension susceptibility | US20090155230 |
| rs4853136 | intergenic | 2 | unknown | hypertension susceptibility | US20090155230 |
| rs6015450 | intergenic | 20 | unknown | systolic hypertension susceptibility diastolic hypertension susceptibility | Nature. 2011 Sep 11;478(7367):103-10 |
| rs632912 | *Metazoa_SRP* | 18 | upstream gene variant | hypertension susceptibility | US20090155230 |
| rs633185 | *ARHGAP42* | 11 | intron variant | systolic hypertension susceptibility diastolic hypertension susceptibility | Nature. 2011 Sep 11;478(7367):103-11 |
| rs6433781 | intergenic | 2 | unknown | hypertension susceptibility | US20090155230 |
| rs6495122 | *CPLX3/ULK3/LMAN1L* | 15 | downstream gene variant | diastolic hypertension susceptibility | Nat Genet. 2009 Jun;41(6):677-87 |
| rs653178 | *ATXN2* | 12 | intron variant | diastolic hypertension susceptibility | Nat Genet. 2009 Jun;41(6):666-76 |
| rs6596140 | intergenic | 5 | unknown | hypertension susceptibility | PLoS One. 2012;7(2):e31489 |
| rs6711736 | intergenic | 2 | unknown | hypertension susceptibility in the young CAD susceptibility | PLoS One. 2009;4(5):e5459;  Eur J Hum Genet. 2012 Mar;20(3):333-40 |
| rs6749447 | *STK39* | 2 | intron variant | ARB response; hypertension | Hypertens Res. 2012 Jan;35(1):107-14 |
| rs6800226 | *FGF12* | 3 | downstream gene variant | hypertension susceptibility | US20090155230 |
| rs6896456 | *LOC100996485* | 5 | intron variant | hypertension susceptibility | US20090155230 |
| rs6940007 | *SLC17A2* | 6 | upstream gene variant | hypertension susceptibility | US20090155230 |
| rs7129220 | *EF537580* | 11 | upstream gene variant | systolic hypertension susceptibility diastolic hypertension susceptibility | Nature. 2011 Sep 11;478(7367):103-13 |
| rs7328290 | intergenic | 13 | unknown | hypertension susceptibility | US20090155230 |
| rs7735940 | intergenic | 5 | unknown | hypertension susceptibility | Hum Genet. 2011 Dec;130(6):725-33 |
| rs7747120 | *GSTA7P* | 6 | downstream gene variant | hypertension susceptibility | US20090155230 |
| rs776746 | *CYP3A5* | 7 | splice acceptor variant | CCB response statin response | Pharmacogenetics. 2004 Aug;14(8):523-5 |
| rs780093 | *GCKR* | 2 | intron variant | hypertension susceptibility triglyceride level | Diabetes. 2011 Apr;60(4):1329-39 |
| rs7827545 | *ZFAT* | 8 | intron variant | hypertension susceptibility | Hum Genet. 2011 Dec;130(6):725-33 |
| rs7984277 | intergenic | 13 | unknown | hypertension susceptibility | US20090155230 |
| rs805303 | *BAG6* | 6 | intron variant | hypertension susceptibility | Nature. 2011 Sep 11;478(7367):103-9 |
| rs901185 | *PIEZO2* | 18 | intron variant | hypertension susceptibility | US20090155230 |
| rs9308945 | intergenic | 2 | unknown | hypertension susceptibility in the young | PLoS One. 2009;4(5):e5459 |
| rs932764 | *PLCE1* | 10 | intron variant | hypertension susceptibility | Nature. 2011 Sep 11;478(7367):103-9 |
| rs9350602 | *MYO6* | 6 | intron variant | hypertension susceptibility | Hum Genet. 2011 Dec;130(6):725-33 |
| rs9586037 | intergenic | 13 | unknown | hypertension susceptibility | US20090155230 |
| rs9618567 | *HIRA* | 22 | intron variant | hypertension susceptibility | US20090155230 |
| rs9815354 | *ULK4* | 3 | intron variant | diastolic hypertension susceptibility | Nat Genet. 2009 Jun;41(6):677-87 |
| rs991316 | intergenic | 4 | unknown | hypertension susceptibility | PLoS Genet. 2009 Jul;5(7):e1000564 |
| rs9951631 | *DSC1* | 18 | intron variant | hypertension susceptibility | US20090155230 |

BMPR1B = Bone morphogenetic protein receptor type-1B; CYP17A1 = cytochrome P450 family 17 subfamily A member 1; GPR39 = G protein-coupled receptor 39; GRK4 = G protein-coupled receptor kinase 4; CACNB2 = calcium voltage-gated channel auxiliary subunit beta 2; PLEKHA7 = pleckstrin homology domain containing A7; CNNM2 = cyclin and CBS domain divalent metal cation transport mediator 2; NT5C2 = 5'-nucleotidase, cytosolic II; KCNH2 = potassium voltage-gated channel subfamily H member 2; ZNF259 = ZPR1 zinc finger; BUD13 = BUD13 homolog; CASZ1 = castor zinc finger 1; PLCD3 = phospholipase C delta 3; SLC39A8 = solute carrier family 39 member 8; UMOD = uromodulin; ZFAT = zinc finger and AT-hook domain containing; CSK = C-terminal Src kinase; MYLK4 = myosin light chain kinase family member 4; LPL = lipoprotein lipase; C10orf107 = ciliary associated calcium binding coiled-coil 1; ZNF652 = zinc finger protein 652; ZNF831 = zinc finger protein 831; FGF5 = fibroblast growth factor 5; MTHFR = methylenetetrahydrofolate reductase; GOSR2 = golgi SNAP receptor complex member 2; HFE = homeostatic iron regulator; MECOM = MDS1 and EVI1 complex locus; TH = tyrosine hydroxylase; CYP3A4 = cytochrome P450 family 3 subfamily A member 4; NR2F2-AS1 = NR2F2 antisense RNA 1; FES = FES proto-oncogene, tyrosine kinase; ATP2B1 = ATPase plasma membrane Ca2+ transporting 1; MOV10 = Mov10 RISC complex RNA helicase; CAPZA1 = capping actin protein of muscle Z-line subunit alpha 1; SH2B3 = SH2B adaptor protein 3; KL = Klotho; MYO6 = myosin VI; PLEKHA7 = pleckstrin homology domain containing A7; ACE = angiotensin I converting enzyme; MECOM = MDS1 and EVI1 complex locus; ARHGAP42 = Rho GTPase activating protein 42; CPLX3 = complexin 3; ULK3 = unc-51 like kinase 3; LMAN1L = lectin, mannose binding 1 like; ATXN2 = ataxin 2; STK39 = serine/threonine kinase 39; FGF12 = fibroblast growth factor 12; SLC17A2 = solute carrier family 17 member 2; GSTA7P = glutathione S-transferase alpha 7, pseudogene; CYP3A5 = cytochrome P450 family 3 subfamily A member 5; GCKR = glucokinase regulator; ZFAT = zinc finger and AT-hook domain containing; BAG6 = BAG cochaperone 6; PIEZO2 = piezo type mechanosensitive ion channel component 2; PLCE1 = phospholipase C epsilon 1; MYO6 = myosin VI; HIRA = histone cell cycle regulator; ULK4 = unc-51 like kinase 4; DSC1 = desmocollin 1; UTR = untranslated region; HDL = high density lipoprotein; CCB = calcium channel blocker; ARB = angiotensin receptor blocker.
