# Supplementary material for: The rs1458038 variant near FGF5 is associated with poor response to calcium channel blockers among Filipinos
Source: Medicine (Baltimore). 2022 Feb 4;101(5):e28703. doi: 10.1097/MD.0000000000028703 (PMC8812666; doi:10.1097/MD.0000000000028703)
Supplement: Supplemental Digital Content [file medi-101-e28703-s002.docx]

**Supplemental Table 2.** Results of the best-case/worst-case analysis for elevated BMI

Best case:

|  | Poor responders | Responders |
| --- | --- | --- |
| Elevated BMI  (≥ 25 kg/m^2^) | 47.69 | 50.00 |
| Non-elevated BMI | 52.31 | 50.00 |
| Total | 100 | 100 |

Pearson’s Χ^2^ = 0.0871

P = 0.768

* Best-case scenario: All poor responders have non-elevated BMI levels, while normal responders have elevated BMI levels.

Worst case:

|  | Poor responders | Responders |
| --- | --- | --- |
| Elevated BMI  (≥ 25 kg/m^2^) | 56.92 | 44.55 |
| Non-elevated BMI | 43.08 | 55.45 |
| Total | 100 | 100 |

Pearson’s Χ^2^ = 2.5046

P = 0.114

* Worst-case scenario: All poor responders have elevated BMI levels, while normal responders have non-elevated BMI levels.
