# Supplementary material for: The rs1458038 variant near FGF5 is associated with poor response to calcium channel blockers among Filipinos
Source: Medicine (Baltimore). 2022 Feb 4;101(5):e28703. doi: 10.1097/MD.0000000000028703 (PMC8812666; doi:10.1097/MD.0000000000028703)
Supplement: Supplemental Digital Content [file medi-101-e28703-s003.docx]

**Supplemental Table 3.** List of variants after genotypic association tests.

| **CHR** | **SNP** | **TEST** | **AFF** | **UNAFF** | **P** |
| --- | --- | --- | --- | --- | --- |
| 4 | rs1458038 | ALLELIC | 64/66 | 47/173 | 1.19E-07 |
| 7 | rs776746 | ALLELIC | 87/43 | 88/132 | 1.67E-06 |
| 4 | rs1458038 | DOM | 46/19 | 38/72 | 4.52E-06 |
| 2 | rs13420028 | ALLELIC | 16/114 | 69/151 | 5.36E-05 |
| 7 | rs776746 | DOM | 56/9 | 63/47 | 5.52E-05 |
| 6 | rs9350602 | ALLELIC | 12/118 | 58/162 | 8.90E-05 |
| 4 | rs991316 | ALLELIC | 14/116 | 63/157 | 9.01E-05 |
| 6 | rs1799945 | ALLELIC | 5/125 | 38/180 | 0.0001496 |
| 8 | rs11780975 | ALLELIC | 4/126 | 35/185 | 0.0001514 |
| 20 | rs16982520 | ALLELIC | 11/119 | 53/167 | 0.0001812 |
| 8 | rs11780975 | DOM | 4/61 | 32/78 | 0.0001894 |
| 1 | rs12046278 | ALLELIC | 53/77 | 48/172 | 0.0002312 |
| 4 | rs991316 | DOM | 11/54 | 48/62 | 0.0002699 |
| 12 | rs653178 | DOM | 12/53 | 49/61 | 0.00052 |
| 12 | rs653178 | ALLELIC | 17/113 | 64/156 | 0.0005989 |
| 6 | rs9350602 | DOM | 10/55 | 44/66 | 0.0006599 |
| 7 | rs776746 | REC | 31/34 | 25/85 | 0.0008055 |
| 15 | rs2398162 | ALLELIC | 48/82 | 122/98 | 0.0008984 |
| 20 | rs16982520 | DOM | 9/56 | 41/69 | 0.0009287 |
| 4 | rs1458038 | REC | 18/47 | 9/101 | 0.0009306 |
| 10 | rs1530440 | ALLELIC | 24/106 | 77/143 | 0.000959 |
| 1 | rs12046278 | DOM | 41/24 | 41/69 | 0.001054 |
| 11 | rs2070762 | ALLELIC | 38/92 | 32/188 | 0.001361 |
| 6 | rs1799945 | DOM | 5/60 | 30/79 | 0.001546 |
| 11 | rs381815 | ALLELIC | 33/97 | 93/127 | 0.001803 |
| 11 | rs381815 | DOM | 23/42 | 66/44 | 0.001834 |
| 2 | rs13420028 | DOM | 15/50 | 52/58 | 0.002094 |
| 9 | rs1110183 | DOM | 49/16 | 57/53 | 0.002334 |
| 2 | rs10188442 | ALLELIC | 9/121 | 41/179 | 0.002444 |
| 11 | rs2070762 | DOM | 31/34 | 28/82 | 0.003088 |
| 8 | rs2954033 | REC | 28/37 | 23/87 | 0.003174 |
| 2 | rs13420028 | REC | 1/64 | 17/93 | 0.003419 |
| 2 | rs6711736 | DOM | 35/30 | 34/76 | 0.003832 |
| 8 | rs2954033 | ALLELIC | 75/55 | 91/129 | 0.00392 |
| 4 | rs13107325 | ALLELIC | 2/128 | 20/200 | 0.005046 |
| 2 | rs6711736 | ALLELIC | 41/89 | 40/180 | 0.005708 |
| 2 | rs10188442 | DOM | 8/57 | 34/76 | 0.005833 |
| 3 | rs448378 | ALLELIC | 36/94 | 93/125 | 0.005866 |
| 2 | rs9308945 | DOM | 49/16 | 60/50 | 0.006334 |
| 4 | rs13107325 | DOM | 2/63 | 18/92 | 0.006654 |
| 8 | rs7827545 | DOM | 30/35 | 29/81 | 0.008553 |
| 12 | rs2681492 | ALLELIC | 45/85 | 108/112 | 0.01023 |
| 10 | rs1530440 | REC | 2/63 | 17/93 | 0.01117 |
| 10 | rs1530440 | DOM | 22/43 | 60/50 | 0.01186 |
| 15 | rs2398162 | DOM | 36/29 | 82/28 | 0.01211 |
| 2 | rs9308945 | ALLELIC | 67/63 | 83/137 | 0.01391 |
| 15 | rs2398162 | REC | 12/53 | 40/70 | 0.01614 |
| 3 | rs448378 | DOM | 29/36 | 69/40 | 0.0184 |
| 8 | rs1372662 | DOM | 29/36 | 29/81 | 0.01955 |
| 13 | rs36217263 | ALLELIC | 41/89 | 45/175 | 0.02139 |
| 1 | rs12046278 | REC | 12/53 | 7/103 | 0.02153 |
| 9 | rs1110183 | ALLELIC | 62/68 | 77/143 | 0.02364 |
| 6 | rs1799945 | REC | 0/65 | 8/101 | 0.02604 |
| 13 | rs36217263 | DOM | 35/30 | 39/71 | 0.02624 |
| 4 | rs2960306 | ALLELIC | 7/123 | 28/192 | 0.02758 |
| 21 | rs13052628 | ALLELIC | 7/123 | 29/191 | 0.0276 |
| 2 | rs780093 | REC | 22/43 | 20/89 | 0.02771 |
| 21 | rs13052628 | DOM | 7/58 | 27/83 | 0.02984 |
| 15 | rs1550576 | ALLELIC | 14/116 | 10/210 | 0.03009 |
| 6 | rs9350602 | REC | 2/63 | 14/96 | 0.03302 |
| 8 | rs2469997 | DOM | 2/63 | 14/96 | 0.03302 |
| 2 | rs6749447 | DOM | 19/46 | 17/93 | 0.03441 |
| 5 | rs6596140 | ALLELIC | 54/76 | 118/102 | 0.0354 |
| 8 | rs1372662 | ALLELIC | 33/97 | 35/185 | 0.03601 |
| 5 | rs7735940 | DOM | 40/25 | 85/25 | 0.03712 |
| 8 | rs7827545 | ALLELIC | 34/96 | 36/184 | 0.03748 |
| 17 | rs16948048 | DOM | 27/38 | 29/81 | 0.04478 |
| 12 | rs2681492 | REC | 10/55 | 32/78 | 0.04504 |
| 12 | rs2384550 | ALLELIC | 23/107 | 22/198 | 0.047 |
| 17 | rs16948048 | ALLELIC | 32/98 | 34/186 | 0.0471 |
| 12 | rs2384550 | DOM | 22/43 | 22/88 | 0.04836 |
| 12 | rs2681492 | DOM | 35/30 | 76/34 | 0.05176 |
| 15 | rs1550576 | DOM | 12/53 | 9/101 | 0.05465 |
| 2 | rs780093 | ALLELIC | 68/62 | 90/128 | 0.05831 |
| 8 | rs2469997 | ALLELIC | 2/128 | 14/206 | 0.06004 |
| 5 | rs6596140 | DOM | 38/27 | 80/30 | 0.06623 |
| 3 | rs448378 | REC | 7/58 | 24/85 | 0.06776 |
| 4 | rs2960306 | DOM | 7/58 | 24/86 | 0.06894 |
| 4 | rs991316 | REC | 3/62 | 15/95 | 0.0722 |
| 3 | rs6800226 | ALLELIC | 64/66 | 86/134 | 0.07385 |
| 1 | rs17367504 | REC | 11/54 | 8/102 | 0.07581 |
| 3 | rs6800226 | REC | 18/47 | 18/92 | 0.08361 |
| 20 | rs16982520 | REC | 2/63 | Dec-98 | 0.08468 |
| 10 | rs1004467 | DOM | 28/37 | 63/47 | 0.08526 |
| 11 | rs2070762 | REC | 7/58 | 4/106 | 0.1026 |
| 20 | rs1327235 | DOM | 43/22 | 86/24 | 0.1092 |
| 2 | rs6749447 | ALLELIC | 20/110 | 21/199 | 0.1215 |
| 18 | rs9951631 | DOM | 14/51 | 36/74 | 0.1228 |
| 10 | rs1004467 | ALLELIC | 37/93 | 81/139 | 0.1284 |
| 18 | rs9951631 | ALLELIC | 15/115 | 39/181 | 0.1288 |
| 7 | rs1137617 | DOM | 11/54 | 30/80 | 0.1411 |
| 10 | rs11191548 | REC | 3/62 | 1/109 | 0.1452 |
| 1 | rs17367504 | ALLELIC | 45/85 | 59/161 | 0.1463 |
| 5 | rs7735940 | ALLELIC | 53/77 | 108/112 | 0.1494 |
| 3 | rs9815354 | ALLELIC | 26/104 | 31/189 | 0.1774 |
| 5 | rs6596140 | REC | 16/49 | 38/72 | 0.1804 |
| 11 | rs381815 | REC | 10/55 | 27/83 | 0.1819 |
| 8 | rs2954033 | DOM | 47/18 | 68/42 | 0.1884 |
| 4 | rs10021303 | ALLELIC | 29/101 | 36/184 | 0.2003 |
| 10 | rs11191548 | ALLELIC | 17/113 | 19/201 | 0.2048 |
| 5 | rs6896456 | DOM | 24/41 | 52/58 | 0.2084 |
| 6 | rs6940007 | DOM | 2/63 | 9/101 | 0.2153 |
| 6 | rs6940007 | ALLELIC | 2/128 | 9/211 | 0.2227 |
| 20 | rs1327235 | ALLELIC | 56/74 | 110/110 | 0.2243 |
| 4 | rs10021303 | DOM | 23/42 | 29/81 | 0.2328 |
| 3 | rs6800226 | DOM | 46/19 | 68/42 | 0.2538 |
| 2 | rs10188442 | REC | 1/64 | 7/103 | 0.2608 |
| 4 | rs17589290 | REC | 1/64 | 6/104 | 0.2608 |
| 8 | rs11780975 | REC | 0/65 | 3/107 | 0.2956 |
| 4 | rs2960306 | REC | 0/65 | 4/106 | 0.2981 |
| 12 | rs653178 | REC | 5/60 | 15/95 | 0.3263 |
| 5 | rs6896456 | ALLELIC | 33/97 | 68/152 | 0.3288 |
| 7 | rs1137617 | ALLELIC | 14/116 | 33/187 | 0.3306 |
| 3 | rs9815354 | REC | 6/59 | 5/105 | 0.3333 |
| 17 | rs12946454 | DOM | 36/29 | 69/41 | 0.3438 |
| 2 | rs9308945 | REC | 18/47 | 23/87 | 0.3568 |
| 2 | rs6711736 | REC | 6/59 | 6/104 | 0.3655 |
| 13 | rs36217263 | REC | 6/59 | 6/104 | 0.3655 |
| 17 | rs12946454 | ALLELIC | 49/81 | 95/125 | 0.3686 |
| 2 | rs6433781 | REC | 1/64 | 0/110 | 0.3714 |
| 12 | rs2384550 | REC | 1/64 | 0/110 | 0.3714 |
| 3 | rs9815354 | DOM | 20/45 | 26/84 | 0.3744 |
| 5 | rs12522034 | ALLELIC | 56/74 | 105/113 | 0.3756 |
| 5 | rs12522034 | DOM | 42/23 | 78/31 | 0.3977 |
| 6 | rs805303 | ALLELIC | 38/92 | 74/146 | 0.4091 |
| 2 | rs780093 | DOM | 46/19 | 70/39 | 0.4095 |
| 6 | rs805303 | REC | 4/61 | Dec-98 | 0.4173 |
| 10 | rs11191548 | DOM | 14/51 | 18/92 | 0.4223 |
| 11 | rs633185 | REC | 8/57 | 9/101 | 0.4318 |
| 13 | rs7328290 | ALLELIC | 34/96 | 66/154 | 0.4648 |
| 4 | rs16998073 | ALLELIC | 47/83 | 88/130 | 0.4953 |
| 4 | rs16998073 | REC | 7/58 | 17/92 | 0.4965 |
| 17 | rs16948048 | REC | 5/60 | 5/105 | 0.5029 |
| 4 | rs13107325 | REC | 0/65 | 2/108 | 0.5304 |
| 21 | rs13052628 | REC | 0/65 | 2/108 | 0.5304 |
| 1 | rs17367504 | DOM | 34/31 | 51/59 | 0.5315 |
| 13 | rs7328290 | REC | 3/62 | 9/101 | 0.5387 |
| 11 | rs633185 | ALLELIC | 40/90 | 60/160 | 0.5406 |
| 4 | rs10021303 | REC | 6/59 | 7/103 | 0.5555 |
| 15 | rs1550576 | REC | 2/63 | 1/109 | 0.5562 |
| 8 | rs15285 | ALLELIC | 4/126 | 10/210 | 0.5833 |
| 13 | rs10492602 | DOM | 5/57 | Dec-95 | 0.6031 |
| 13 | rs10492602 | ALLELIC | 5/119 | 12/202 | 0.613 |
| 5 | rs1173771 | REC | 8/57 | Nov-99 | 0.6251 |
| 4 | rs17589290 | DOM | 26/39 | 40/70 | 0.6326 |
| 15 | rs6495122 | DOM | 36/29 | 55/53 | 0.638 |
| 6 | rs805303 | DOM | 34/31 | 62/48 | 0.6392 |
| 13 | rs7328290 | DOM | 31/34 | 57/53 | 0.6406 |
| 2 | rs6749447 | REC | 1/64 | 4/106 | 0.6523 |
| 7 | rs1137617 | REC | 3/62 | 3/107 | 0.6718 |
| 9 | rs16931920 | DOM | 3/62 | 3/107 | 0.6718 |
| 9 | rs16931920 | ALLELIC | 3/127 | 3/217 | 0.6744 |
| 2 | rs6433781 | ALLELIC | 13/117 | 19/201 | 0.7032 |
| 17 | rs12946454 | REC | 13/52 | 26/84 | 0.7074 |
| 5 | rs12522034 | REC | 14/51 | 27/82 | 0.7134 |
| 15 | rs6495122 | ALLELIC | 44/86 | 69/147 | 0.7239 |
| 4 | rs16998073 | DOM | 40/25 | 71/38 | 0.7445 |
| 6 | rs7747120 | DOM | 3/62 | 7/103 | 0.7462 |
| 6 | rs7747120 | ALLELIC | 3/127 | 7/213 | 0.7499 |
| 11 | rs633185 | DOM | 32/33 | 51/59 | 0.7554 |
| 8 | rs15285 | DOM | 4/61 | 9/101 | 0.7695 |
| 5 | rs1173771 | ALLELIC | 37/93 | 60/160 | 0.8062 |
| 10 | rs11014166 | DOM | 8/57 | 16/94 | 0.8211 |
| 10 | rs1004467 | REC | 9/56 | 18/92 | 0.8289 |
| 10 | rs11014166 | ALLELIC | 9/121 | 17/203 | 0.8362 |
| 2 | rs6433781 | DOM | 12/53 | 19/91 | 0.8404 |
| 9 | rs1110183 | REC | 13/52 | 20/90 | 0.8421 |
| 20 | rs1327235 | REC | 13/52 | 24/86 | 0.8495 |
| 4 | rs17589290 | ALLELIC | 27/103 | 46/174 | 1 |
| 5 | rs1173771 | DOM | 29/36 | 49/61 | 1 |
| 5 | rs7735940 | REC | 13/52 | 23/87 | 1 |
| 5 | rs6896456 | REC | 9/56 | 16/94 | 1 |
| 6 | rs6940007 | REC | 0/65 | 0/110 | 1 |
| 6 | rs7747120 | REC | 0/65 | 0/110 | 1 |
| 8 | rs15285 | REC | 0/65 | 1/109 | 1 |
| 8 | rs2469997 | REC | 0/65 | 0/110 | 1 |
| 8 | rs7827545 | REC | 4/61 | 7/103 | 1 |
| 8 | rs1372662 | REC | 4/61 | 6/104 | 1 |
| 9 | rs16931920 | REC | 0/65 | 0/110 | 1 |
| 10 | rs11014166 | REC | 1/64 | 1/109 | 1 |
| 11 | rs7129220 | ALLELIC | 4/126 | 6/214 | 1 |
| 11 | rs7129220 | DOM | 4/61 | 6/104 | 1 |
| 11 | rs7129220 | REC | 0/65 | 0/110 | 1 |
| 13 | rs10492602 | REC | 0/62 | 0/107 | 1 |
| 15 | rs6495122 | REC | 8/57 | 14/94 | 1 |
| 18 | rs9951631 | REC | 1/64 | 3/107 | 1 |

CHR = chromosome; SNP = single nucleotide polymorphism; AFF = affected; UNAFF = unaffected; DOM = dominant; REC = recessive.
