# Supplementary material for: The rs1458038 variant near FGF5 is associated with poor response to calcium channel blockers among Filipinos
Source: Medicine (Baltimore). 2022 Feb 4;101(5):e28703. doi: 10.1097/MD.0000000000028703 (PMC8812666; doi:10.1097/MD.0000000000028703)
Supplement: Supplemental Digital Content [file medi-101-e28703-s004.docx]

**Supplemental Table 4.** Power analysis of the study.

| Case-control statistics: general 2 df test  (BB versus Bb versus bb)  Sample NCP = 10.56  **Alpha** | **Power** | **N cases for 80% power** |
| --- | --- | --- |
| 0.1 | 0.9027 | 47 |
| 0.05 | 0.8371 | 59 |
| 0.01 | 0.6469 | 85 |
| 0.001 | 0.3739 | 121 |
| *0.00081* | 0.3524 | 124 |

*df,* degrees of freedom

For the study to be 80% powered at an adjusted alpha of 0.00081, the minimum number of cases must be 124.
